# Growth or immunity? OsCBSX3’s molecular toggle decides

**DOI:** 10.1007/s44297-025-00057-0

**Published:** 2025-08-13

**Authors:** Mengying Pu, Shan Liu, Yanjie Xie, Jian Chen

**Affiliations:** 1 International Genome Center, Jiangsu University, Zhenjiang, China; 2 Co-Innovation Center for Sustainable Forestry in Southern China, State Key Laboratory of Tree Genetics and Breeding, Key Laboratory of State Forestry and Grassland Administration on Subtropical Forest Biodiversity Conservation, College of Life Sciences, Nanjing Forestry University, Nanjing, Jiangsu China; 3 Laboratory Center of Life Sciences, College of Life Sciences, Nanjing Agricultural University, Nanjing, China

Plants must dynamically balance growth and defense to adapt to a wide array of biotic and abiotic stresses. However, constitutive or excessive defense activation often comes at the cost of plant growth and development, which is described as a “growth-defense trade-off”. For instance, a recent study showed that knockout of phosphatidate phosphohydrolase genes confers wide‐spectrum disease resistance in rice, but leads to significant growth retardation compared to wild-type plants [5].

“Resource constraint hypothesis” is one of the most prevailing explanations for the underlying mechanisms governing growth-defense trade-offs. This hypothesis posits that plant resources are finite, and reallocating these resources towards defense may directly diminish the energy or resources available for growth and developmental processes, owing to the inherent resource limitations in plants [6]. However, accumulating evidence indicates that plants may actively regulate growth-defense trade-offs via a sophisticated multi-layered regulatory network, with plant hormone crosstalk playing a major role in this network [4, 7, 16]. In addition, molecules such as microRNAs and non-long coding RNAs, epigenetic regulation as well as post-translational modifications of major transcriptional factors and Nucleotide-binding Leucine-rich Repeat Receptor (NLR) are also involved in modulating growth-defense trade-offs [6, 12, 13, 15].

As a gaseous signaling molecule, hydrogen sulfide (H_2_S) acts as a pivotal regulator not only in plant growth and development but also in responses to various abiotic and biotic stresses [2]. To maintain plant growth homeostasis, H_2_S promotes seed germination by activating antioxidant enzymes and MAPK signaling pathways [8]. In addition, H_2_S enhances photosynthetic efficiency and senescence resistance through upregulating photosynthetic enzymes, light signaling responses, and suppressing chlorophyll degradation genes [1, 10]. Under unfavorable abiotic stresses such as heavy metal, drought, heat, cold and salinity, H_2_S promotes stress resilience by enhancing antioxidant enzyme activities, photosynthetic system protection, sulfur metabolism regulation and interacting with other signalling molecules such as phytohormones [8, 11, 14, 18]. In addition, H_2_S is also involved in governing plant defense responses against pathogens and herbivores by inducing the expression of pathogenesis-related genes and other defense-related genes, regulating glutathione metabolism and enzyme activity, as well as interacting with phytohormones [3, 14]. However, to date, limited research has addressed the role of H_2_S in coordinating plant growth-defense trade-offs, and the signaling networks governing this crosstalk are yet to be elucidated.

In an exciting recent study, Zhang et al. [17] found that H_2_S plays a dual role in balancing rice immunity and development. Exogenous H_2_S application improved rice resistance to *Xanthomonas oryzae* pv. *oryzicola (Xoc)* and *X. oryzae* pv. *Oryzae (Xoo)* by inducing H_2_O_2_ release, ROS burst and defense gene expression. However, it also negatively affected rice growth, specifically in terms of root elongation and the development of lateral roots. The mechanisms of this phenomenon were found to be attributed to the protein state conversion of a CBS (Cystathionine β-synthase) domain-containing protein OsCBSX3, a homolog of H_2_S synthesis-related protein in mammals, which catalyzes the condensation of cysteine and homocysteine, forming cystathionine and H_2_S [9]. The authors showed that OsCBSX3 positively regulates rice resistance to *Xoc* and *Xoo* due to its ability to produce H_2_S. Like other CBS-domain containing proteins, OsCBSX3 protein exists in two different states in rice, monomer and oligomer. Bacterial infection (*Xoc* and *Xoo*) leads to the shift of monomeric OsCBSX3 to an oligomeric form, which can enter into chloroplast. The oligomerization-deficient mutant showed impaired H_2_S production, compromised resistance against *X. oryzae*, and defective chloroplast localization, highlighting the essential role of oligomeric OsCBSX3 in plant defense to pathogens.

To explore how OsCBSX3-mediated H_2_S production is involved in regulating rice growth-defense trade-offs, the authors identified two OsCBSX3-interacting proteins OsTrxZ and OsPsbO. TrxZ is a member of thioredoxin, while PsbO is the photosystem II (PSII) manganese-stabilizing protein. They found that PsbO binds to OsCBSX3 upon pathogen infection, resulting in elevated H_2_S generation and consequently enhanced disease resistance in rice. Once H_2_S level is exceeded, PsbO-mediated monomer-to-oligomer transition of OsCBSX3 will be compromised by TrxZ’s competitive binding to OsCBSX3, mediating reduction of OsCBSX3 to monomers and alleviating overaccumulation of H_2_S as well as its detrimental impacts on rice growth and development (Fig. 1). Together, these results indicate that the TrxZ/PsbO involved redox-mediated OsCBSX3 protein state transition balances rice growth and defense by fine-tuning biosynthesis of H_2_S in rice.

**Fig. 1:**
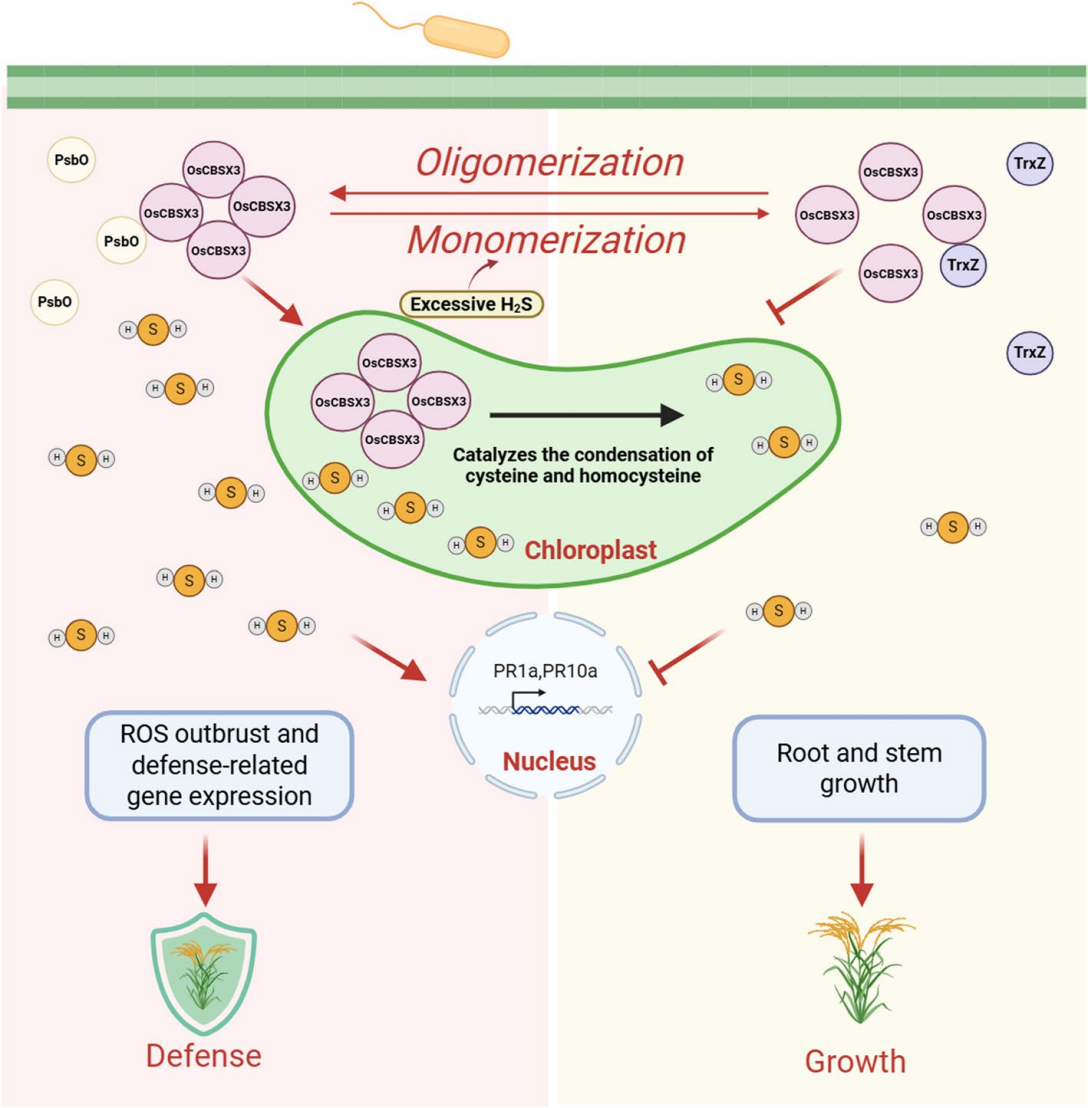
OsTrxZ–OsCBSX3–PsbO module-mediated H_2_S synthesis regulates growth-immunity balance in rice. Upon bacterial infection, PsbO facilitates the oligomerization of OsCBSX3, leading to the synthesis of H_2_S, which subsequently induces bursts of reactive oxygen species (ROS) and amplifies the activation of defense-related genes against pathogenic bacteria. Excessive H_2_S, in turn, promotes the interaction between OsTrxZ and OsCBSX3, resulting in the monomerization of OsCBSX3, which reduces H_2_S levels and assists in rice growth and development

This study, for the first time, sheds light on the role of H_2_S in coordinating plant growth-defense trade-offs and the underlying mechanisms. It also initially reveals that OsCBSX3, a CBS structural domain protein, catalyzes H_2_S synthesis in plants. On the one hand, this discovery renews the conventional notion that H_2_S is exclusively produced by cysteine desulfurization enzymes including L-cysteine desulfhydrase (LCD) and D-cysteine desulfhydrase (DCD), filling the gaps in terms of CBS-mediated H_2_S synthesis pathways in plants [11]. On the other hand, it clarifies the universal strategy of the “growth-defense trade-off” via the dynamic regulation of metabolic enzyme activities of OsCBSX3. More importantly, by uncovering molecular mechanisms underlying H_2_S-meidated growth-defense balancing in rice, this study provides multiple potential targets, including OsCBSX3, PsbO and OsTrxZ, for rice disease resistance breeding. Notably, H_2_S concentration thresholds need to be optimized to avoid excessive growth inhibition and achieve “on-demand disease resistance”. This can be achieved through precise gene editing techniques, including the use of pathogen-inducible promoters.

## Data Availability

Not applicable.
